# 

**Published:** 1887-11-26

**Authors:** 


### CONTENTS.

**Fingers and Brains** . . . . . . . . 141

**Words of Consolation.**—Chapter LX. The Sufferings of Christ . . . 142

**Female Medical Aid to the Women of India.** By The Countess of Dufferin, Lady President of the National Association . . . . 143

**The Editor's Letter-Box** . . . . . 143

**The Doctor's Pulpit.**—Doctors and Patients . . . . 144

**From Friends Across the Sea.** By our Readers' Relations and Colonial Cousins . . . . 145

**The Sick, the Churches, and the Hospitals** . . 145

**Turning over New Leaves.**—Pulmonary Consumption.—Mr. Besant's New Book . . . . 146

**Professional Notes.** By Geo. W. Potter, M.D. . . 147

**National Pension Fund** . . . . . 148

**Notes and News.**—The Parkes Museum.—Mr. G. Herring's Jubilee Offering for the North-West London Hospital.—Meeting of the Medico-Psychological Association at Edinburgh. — Montrose Royal Asylum. — University College Hospital and Mr. Quain's Legacy.—Vacancies, etc., etc. . 148

**Annotations.**—The Science of Superstition.—A Salt Water Supply for Edinburgh.—The Phonograph . . 151

**The Common Accidents of Every-day Life.** By Robt. A. B. Forrester, B.A. . . . . . 152

**Cottage Hospitals** . . . . . . 152

**East and West.** By Leslie Keith. Chapter IX. Facing the Question . . . . . . 153

**Medicine Men of the World.** By the Man in the Crowd. Chapter IV. Savage and Civilised Practitioners . . 155

**The Hospitals Association.**—A Discussion on Hospital Construction . . . . . . 156

**Amusements with Profit for all Ages** . . . 157

